# Enhancement of ionospheric heating effect by chemical release

**DOI:** 10.1038/s41598-024-64011-w

**Published:** 2024-06-09

**Authors:** Hai-Sheng Zhao, Jie Feng, Zheng-Wen Xu, Ya-Xin Liu, Kun Xue, Jian Wu, Cheng Wang, Shou-Zhi Xie, Huai-Yun Peng

**Affiliations:** 1 National Key Laboratory of Electromagnetic Environment, China Research Institute of Radiowave Propagation, Qingdao, 266107 China; 2 Kunming Electro-Magnetic Environment National Observation and Research Station, China Research Institute of Radiowave Propagation, Qujing, 655500 China; 3 Qian Xuesen Laboratory of Space Technology, China Academy of Space Technology, Beijing, 100094 China

**Keywords:** High frequency pump wave, Heating, Low ionosphere, Absorption mitigation, Space physics, Physics

## Abstract

The ionosphere can be artificially modified by employing ground-based high-power high-frequency electromagnetic waves to irradiate the ionosphere. This modification is achieved through the nonlinear interaction between the electromagnetic waves and the ionospheric plasma, leading to changes in the physical properties and structure of the ionosphere. The degree of artificial modification of the ionosphere is closely related to the heating energy density of high-frequency pump waves. Due to the high density of neutral constituents in the lower ionosphere and the high frequency of electron-neutral collisions, the energy of heating pump waves will be absorbed and attenuated during the penetration of the low ionosphere, seriously affecting the heating effect. This paper proposes a method to reduce the absorption of ionospheric heating pump waves by releasing electron attachment chemicals into low ionosphere to form a large-scale electron density hole. A model for mitigating pump waves absorption based on SF_6_ release is established, and the absorption at different frequencies is quantitatively calculated. The propagation characteristics of high-frequency signals in ionospheric holes are studied using a three-dimensional ray tracing method, and the results demonstrate that the chemical release method not only reduces the absorption attenuation of heating pump waves but also forms spherical electron density holes, which exhibit a focusing effect on the heating beam and enhance the heating effect. The results are of great significance for understanding the nonlinear interaction between electromagnetic wave and ionospheric plasma and improving the ionospheric heating efficiency.

## Introduction

Since the discovery of the Luxembourg Effect^1^ in the 1930s, active modification of the space environment has become a research focus for countries worldwide. Using ground-based radio transmitters, high-power, high-frequency radio waves are emitted into the ionosphere. Through the nonlinear interaction with ionospheric plasma, it excites various parametric instabilities, and then alter the distribution of electron temperature and density in the heated region through processes like thermal convection and diffusion. This, in turn, generates localized structures of electron density irregularities aligned along the geomagnetic field and other nonlinear perturbations that alter the physical properties of the local ionosphere, collectively referred to as “ionospheric heating”^2–7^. In the 1970s, the first ionospheric heating experimental facility was constructed and operated in Platteville, Colorado, USA, yielding a series of remarkable achievements^8^. In view of the characteristics of the ionosphere as a natural plasma laboratory, as well as the potential application of various nonlinear effects observed during ionospheric heating, Western countries, led by the United States, established multiple high-power high-frequency (HF) transmitters to investigate the nonlinear processes involved in ionospheric heating, thereby promoting the development of the ionospheric plasma physics research. These ionospheric heating facilities are predominantly located in high-latitude regions of the Northern Hemisphere, including the Sura heating station constructed by the former Soviet Union^9^, the HIPAS and HAARP stations in Alaska^10,11^, USA, and the Tromsø station in Norway^12,13^. Additionally, there are two stations situated in mid-to-low latitude regions: the Platteville station in Colorado and the Arecibo station in Puerto Rico^14,15^.

The ionospheric plasma is a time-varying medium exhibiting distinct spatiotemporal characteristics, and its heating effects demonstrate features related to solar activity, seasons, and diurnal variations. The ionospheric heating effect is stable during nighttime; however, during the pre-sunrise and midday periods, the effective radiated power reaching the heating altitudes is reduced due to the absorption effects in the low ionosphere. This results in lower trigger efficiency and poorer heating experimental effect^16,17^.

This study addresses the demand for mitigating the absorption effects of high-frequency radio wave heating in the lower ionosphere. A large number of studies and experiments have been carried out on ionospheric hole and electron density irregularities caused by the chemical release^18–21^. However, there are no relevant reports on the use of electrons attachment by chemical release to achieve low ionospheric absorption reduction of heat pump beam. Hence, we propose a method to mitigate the absorption of ionospheric heating pump waves by releasing electron attachment chemicals at low ionospheric altitudes. A model for mitigating ionospheric heating absorption based on SF_6_ release is established, and the absorption of pump waves at different frequencies is quantitatively calculated. The propagation characteristics of high-frequency signals in ionospheric holes are studied using a three-dimensional ray tracing method, and the results demonstrate that the chemical release method not only reduces the absorption attenuation of heating pump waves but also forms spherical electron density holes, which exhibit a focusing effect on the heating beam and enhance the heating effect. The research of this study provides important theoretical support for studying the nonlinear interaction processes between electromagnetic waves and ionospheric plasma and improving the efficiency of ionospheric heating.

## The low ionospheric chemical release theory

Ionospheric chemical release refers to the injection of chemicals into ionospheric altitudes using sounding rockets, satellites, and spacecraft, among other launch vehicles. This artificial manipulation modifies the composition and structure of the ionospheric plasma, resulting in significant short-term changes in the ionosphere^22–25^. The release of ionospheric chemicals holds significant importance for the in-depth investigations into the dynamics and coupling mechanisms of the ionospheric system, the study of ionospheric instability excitation mechanisms and processes, and the construction of a new instability theory system.

### Chemicals diffusion

In the initial stage of chemical release, under the influence of pressure, the release pushes the surrounding plasma away like a snowplow. This process occurs at supersonic speeds and lasts only a few seconds. Subsequently, the pressure difference rapidly decreases until it becomes comparable to the background pressure. At this point, the released material and the surrounding plasma mix thoroughly and begin to diffuse into space. This diffusion process takes a long time and in which the ionic chemical reaction mainly occurs.

The diffusion equation is expressed as:

1 $$\frac{{\partial n_{i} }}{\partial t} = D[\frac{{\partial^{2} n_{i} }}{{\partial x^{2} }} + \frac{{\partial^{2} n_{i} }}{{\partial y^{2} }} + \frac{{\partial^{2} n_{i} }}{{\partial z^{2} }}]$$

where $D$ is the diffusion coefficient, $n_{i} (x,y,z,t)$ is the number density of releases and satisfies $n_{i} (x,y,z,0) = N_{0} \delta (x,y,z)$, $N_{0}$ is the total number of molecules released.

Considering the release at the beginning of the release as a point source. Under the assumption of a stratified background ionosphere and thermosphere, the diffusion process of the released material can be approximated by the following equation^23^:

2 $$\begin{gathered} n_{i} (r,z,t) = \frac{{N_{0} }}{{(4\pi D_{0} t)^{1.5} }}\exp \{ - (z - z_{0} )(\frac{3}{{4H_{\alpha } }} + \frac{1}{{2H_{i} }}) - \alpha t \hfill \\ \begin{array}{*{20}c} {} & {} & {} \\ \end{array} - \frac{{H_{\alpha }^{2} \{ 1 - \exp [ - (z - z_{0} )/(2H_{\alpha } )]\}^{2} }}{{4D_{0} t}} - \frac{{r^{2} \exp [ - (z - z_{0} )/(2H_{\alpha } )]}}{{4D_{0} t}} \hfill \\ \begin{array}{*{20}c} {} & {} & {} \\ \end{array} - (\frac{1}{{H_{\alpha } }} - \frac{1}{{H_{i} }})^{2} \frac{{D_{0} t\exp [(z - z_{0} )/(2H_{\alpha } )]}}{4}\} \hfill \\ \end{gathered}$$

where $n_{i} (r,z,t)$ is the density of released material as a function of time t and space (r and z are the radial distance from the point source and the altitude of the ionosphere, respectively), $z_{0}$ is the altitude of the point of release, $N_{0}$ is the total number of molecules released, and $D_{0}$ is the release point diffusion coefficient. $H_{\alpha } = kT/m_{a} g$ is the atmospheric scale altitude and $H_{i} = kT/m_{i} g$ is the released gas scale altitude, where $k$ is the Boltzmann constant, $T$ is the neutral gas temperature, $m_{a}$ and $m_{i}$ are the average molecular weight of the atmosphere and the molecular weight of the released gas, respectively, $g$ is the gravitational acceleration, and $\alpha t$ is the loss term due to chemical reactions.

### Chemical reaction of releases with the ionosphere

The main chemical reaction processes of SF_6_ in the ionospheric plasma are shown in Table 1. Among the four equations in Table 1, the reaction efficiency of Eqs. (3) and (4) is very low due to the low O^+^ density at 100 km and 120 km altitude, and they belong to the secondary reactions. In Eqs. (1) and (2), Eq. (2) is more important than Eq. 1, because the reaction coefficient k_2_ of Eq. (2) is much larger than k_1_.

**Table 1:** Reactions stimulated by SF_6_ release in ionosphere^26^.

Index	Reaction equation	Reaction rate (cm^3^/s)
1	$SF_{6} + e^{ - } \underset{{k_{a} }}{\overset{{k_{1} }}{\longleftrightarrow}}\left( {SF_{6}^{{^{ - } }} } \right)^{*} \mathop{\longrightarrow}\limits^{{k_{b} }}SF_{6}^{ - }$	$$\begin{gathered} k_{1} = {{2.2 \times 10^{ - 7} } \mathord{\left/ {\vphantom {{2.2 \times 10^{ - 7} } {\left[ {1 + 640\,\exp \left( { - 4770/T} \right)} \right]}}} \right. \kern-0pt} {\left[ {1 + 640\,\exp \left( { - 4770/T} \right)} \right]}} \hfill \\ k_{a} = 4.00 \times 10^{4} \,s^{ - 1} \hfill \\ k_{b} = {{k_{a} } \mathord{\left/ {\vphantom {{k_{a} } {10}}} \right. \kern-0pt} {10}} \hfill \\ \end{gathered}$$
2	$SF_{6} + e^{ - } \mathop{\longrightarrow}\limits^{{k_{2} }}SF_{5}^{ - } + F$	$k_{2} = 2.2 \times 10^{ - 7} - k_{1}$
3	$SF_{6}^{ - } + O^{ + } \mathop{\longrightarrow}\limits^{{k_{5} }}SF_{6} + O^{*}$	$k_{5} \approx 5 \times 10^{ - 8}$
4	$SF_{5}^{ - } + O^{ + } \mathop{\longrightarrow}\limits^{{k_{6} }}SF_{6} + O$	$k_{6} \approx k_{5}$

### Plasma diffusion

The change in electron density in the release region of ionospheric chemicals disrupts the existing density distribution structure and dynamic equilibrium of charged particles. According to plasma diffusion theory, the transport equation for plasma can be derived as follows^27^:

3 $$\begin{gathered} \frac{{\partial n_{p} }}{\partial t} = \sin^{2} I\left( {\frac{\partial D}{{\partial z}}\frac{{\partial n_{p} }}{\partial z} + D\frac{{\partial^{2} n_{p} }}{{\partial z^{2} }} + \frac{{n_{p} }}{{T_{p} }}\frac{\partial D}{{\partial z}}\frac{{\partial T_{p} }}{\partial z} + \frac{D}{{T_{p} }}\frac{{\partial n_{p} }}{\partial z}\frac{{\partial T_{p} }}{\partial z} - \frac{{Dn_{p} }}{{T_{p}^{2} }}\frac{{\partial^{2} T_{P} }}{{\partial z^{2} }} + \frac{{Dn_{p} }}{{T_{P} }}\frac{{\partial^{2} T_{P} }}{{\partial z^{2} }}} \right) \hfill \\ \begin{array}{*{20}c} {} & {} \\ \end{array} + \sin I\cos I\left( {\frac{\partial D}{{\partial z}}\frac{{\partial n_{p} }}{\partial x} + D\frac{{\partial^{2} n_{p} }}{\partial x\partial z}} \right) + \sin I\sin \gamma \left( {\frac{\partial D}{{\partial z}}\frac{{\partial n_{p} }}{\partial y} + D\frac{{\partial^{2} n_{p} }}{\partial y\partial z}} \right) \hfill \\ \begin{array}{*{20}c} {} & {} \\ \end{array} + \sin^{2} I\left( {\frac{{n_{p} }}{{H_{p} }}\frac{\partial D}{{\partial z}} + \frac{D}{{H_{p} }}\frac{{\partial n_{p} }}{\partial z} - \frac{{Dn_{p} }}{{H_{P}^{2} }}\frac{{\partial H_{p} }}{\partial z}} \right) - v_{D} \cdot \sin I\frac{{\partial n_{p} }}{\partial z} \hfill \\ \begin{array}{*{20}c} {} & {} \\ \end{array} + \sin I\cos I\left( {D\frac{{\partial^{2} n_{p} }}{\partial x\partial z} + \frac{D}{{T_{p} }}\frac{{\partial n_{p} }}{\partial x}\frac{{\partial T_{p} }}{\partial z}} \right) + D \cdot \cos^{2} I\frac{{\partial^{2} n_{p} }}{{\partial x^{2} }} + D \cdot \cos I \cdot \sin \gamma \frac{{\partial^{2} n_{p} }}{\partial y\partial x} \hfill \\ \begin{array}{*{20}c} {} & {} \\ \end{array} + \sin I\cos I\left( {\frac{D}{{H_{p} }}\frac{{\partial n_{p} }}{\partial y} - \frac{{Dn_{p} }}{{H_{P}^{2} }}\frac{{\partial H_{p} }}{\partial x}} \right) - v_{D} \cdot \cos I\frac{{\partial n_{p} }}{\partial x} \hfill \\ \begin{array}{*{20}c} {} & {} \\ \end{array} + \sin \gamma \sin I\left( {D\frac{{\partial^{2} n_{p} }}{\partial z\partial y} + \frac{D}{{T_{p} }}\frac{{\partial n_{p} }}{\partial y}\frac{{\partial T_{p} }}{\partial z}} \right) + D \cdot \sin \gamma \cos I\frac{{\partial^{2} n_{p} }}{\partial x\partial y} + D \cdot \sin^{2} \gamma \frac{{\partial^{2} n_{p} }}{{\partial y^{2} }} \hfill \\ \begin{array}{*{20}c} {} & {} \\ \end{array} + \sin \gamma \sin I\left( {\frac{D}{{H_{p} }}\frac{{\partial n_{p} }}{\partial y} - \frac{{Dn_{p} }}{{H_{P}^{2} }}\frac{{\partial H_{p} }}{\partial y}} \right) - v_{D} \cdot \sin \gamma \frac{{\partial n_{p} }}{\partial y} + P_{p} - L_{p} \hfill \\ \end{gathered}$$

where $n_{p}$ is the density of ions or electrons; $P_{p}$ and $L_{p}$ represent the production and recombination rates of charged particles, respectively. $L_{p} = L_{0} + \sum\limits_{i = 1}^{M} {\,K_{1i} n_{p} n_{i} }$, where $L_{0}$ is the loss rates of $O^{ + }$ reacting with other particles and photochemical reactions, the released neutral gas consists of M species of neutral molecules, and $n_{i}$ represents the chemical reaction rate between species $i$ and $O^{ + }$; $T_{p} = (T_{e} + T_{i} )/2$ is the plasma temperature; $H_{p}$ is the plasma scale altitude , $H_{p} = 2T_{p} k/(m_{p} g)$; $I$ is the magnetic inclination angle, $\gamma$ is the magnetic declination Angle; $D$ is the effective bipolar diffusion coefficient, $D = (1 + T_{e} /T_{i} )D_{i}$, where $D_{i}$ is the ion diffusion coefficient; $v_{D}$ represents the applied drift velocity (wind speed).

## Numerical simulation of ionospheric heating absorption attenuation

### Simulation of ionospheric depletion region generation and evolution

**Figure 1:**
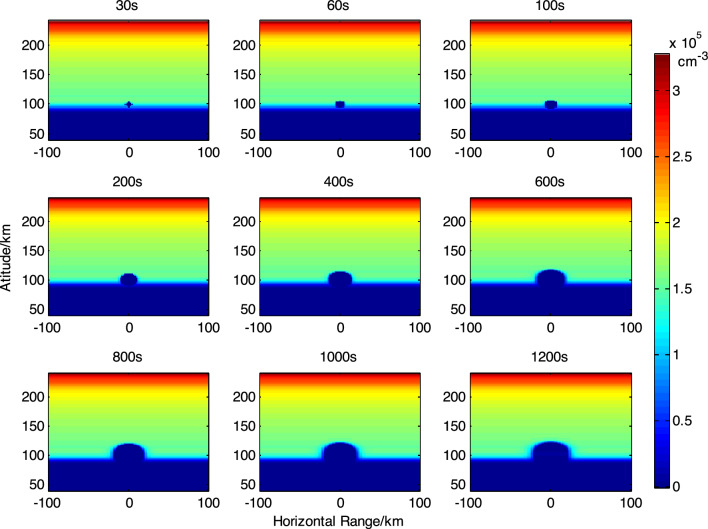
Evolution of the effect of releasing 30 kg SF_6_ at 100 km altitude.

**Figure 2:**
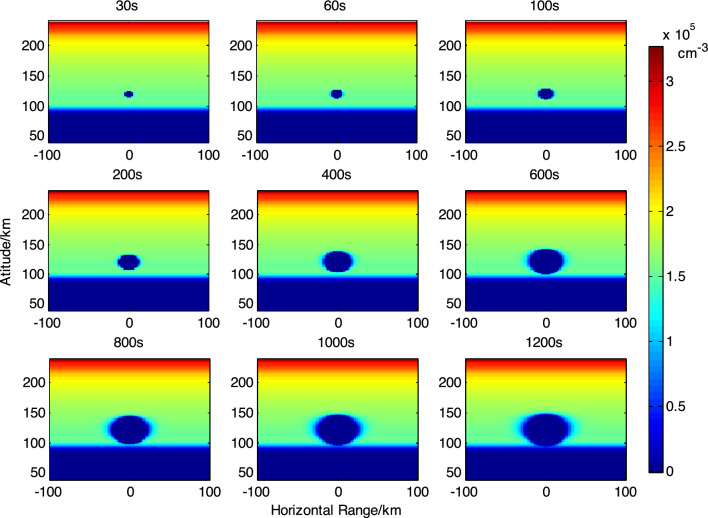
Evolution of the effect of releasing 30 kg SF_6_ at 120 km altitude.

In order to study the method of eliminating the absorption of high frequency radio waves in the lower ionosphere by releasing electron-attached chemicals, the background ionosphere generated by IRI2020 model at 12:00 local time on April 15, 2022 in Hainan region was numerically simulated. The ionospheric disturbance effects resulting from the release of 30 kg of SF_6_ at altitudes of 100 km and 120 km were simulated. As shown in Fig. 1 and Fig. 2.

From the simulation results, it can be observed that the maximum ionospheric hole diameter formed by the release of 30 kg SF_6_ at an altitude of 100 km is approximately 25 km. The maximum ionospheric hole diameter formed by releasing 30 kg SF_6_ at an altitude of 120 km is about 30 km, and the electron density is close to complete depletion. It should be noted that when releasing chemicals at low ionospheric altitudes, the smaller diffusion coefficient of the released material results in smaller ionospheric hole scales, requiring higher control of the release point in space experiments.

### Calculation of low ionospheric absorption attenuation for typical time periods

According to the absorption attenuation theory, the total absorption attenuation L of the lower ionosphere can be expressed as^28^:

4 $${\text{L = }}\frac{{e^{2} }}{{2\varepsilon_{0} mcu}}\frac{{n_{e} v}}{{w^{2} + v^{2} }}S$$

where $n_{e}$ is the electron density, $\varepsilon_{0}$ is the vacuum permittivity, $c$ is the speed of light, $\mu$ is the magnetic permeability, $m$ is the electron mass, $e$ is the elementary charge, $v$ is the collision frequency, $w$ is the incident wave frequency, and $S$ is the propagation distance.

The collision frequency $v$ can be expressed as^29^:

5 $$v = \frac{{5.45 \times 10^{ - 5} n}}{{T^{3/2} }}$$

where $n$ is the neutral component density (cm^−3^) and $T$ is the temperature.

**Figure 3:**
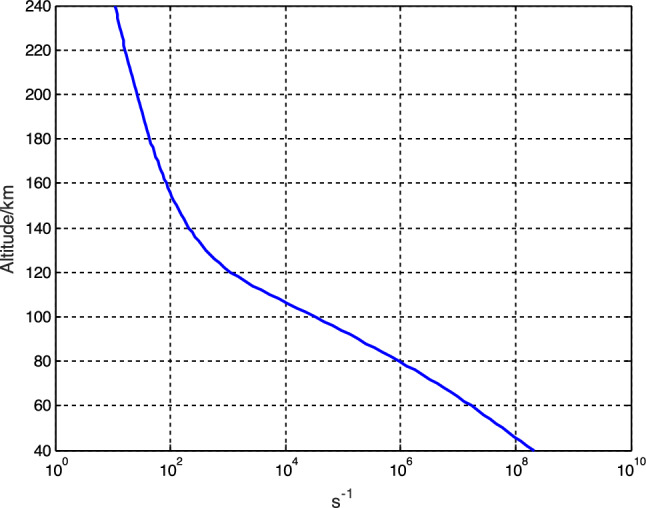
Electron-neutral component collision frequency profiles.

According to the atmosnrlmsise00 model, the altitude profiles of neutral component density and temperature are calculated. The following seven neutral molecules are considered in the calculation process: He, O, N_2_, O_2_, Ar, H and N. Based on this, the profile of electron-neutral molecule collision frequency is computed, as depicted in Fig. 3.

**Figure 4:**
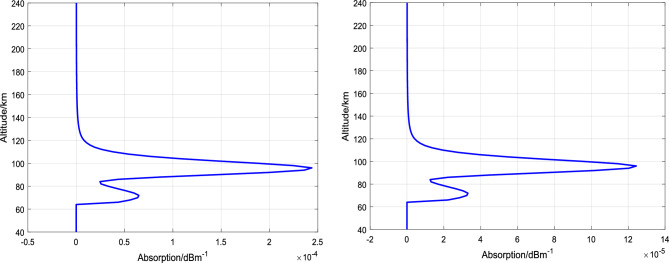
Variation of incident wave absorption coefficient with altitude (left: 5 MHz, right: 7 MHz).

Based on the theory of absorption attenuation in the low ionosphere and the above analyses, the altitude distribution of absorption coefficients at 5 MHz and 7 MHz is calculated as shown in Fig. 4. By integrating the absorption coefficients over altitude, the total absorption attenuation is 4.77 dB for 5 MHz and 2.43 dB for 7 MHz in the altitude range of 40 − 240 km. The maximum absorption altitude varies under different ionospheric backgrounds (different locations, seasons, solar activity) and heating frequencies. To enhance the heating effect using electron-captured material release methods, the release altitude of the material needs to be optimized based on the ionospheric background conditions and heating frequency.

### Evaluation of absorption elimination effect in the low ionosphere

**Figure 5:**
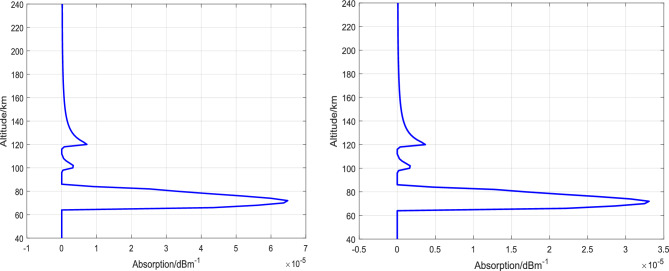
Variation of incident wave absorption coefficient with altitude (600 s-100 km-30 kg SF_6_, left: 5 MHz, right: 7 MHz).

At an altitude of 100 km, after a time period of 600 s following the release, the total absorption attenuation is calculated to be 1.04 dB for the 5 MHz incident wave and 0.53 dB for the 7 MHz incident wave in the altitude range of 40–240 km. The distribution of absorption attenuation coefficients is illustrated in Fig. 5.

**Figure 6:**
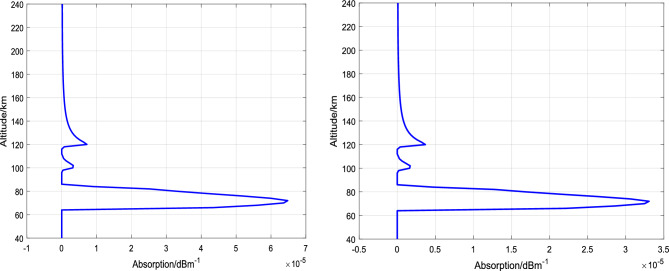
Variation of incident wave absorption coefficient with altitude (600 s-120 km-30 kg SF_6_, left: 5 MHz, right: 7 MHz).

At an altitude of 120 km, 600 s after release, using the same calculation method for absorption coefficients as mentioned above, the total absorption of 5 MHz incident wave is calculated to be 4.16 dB, and the total absorption of 7 MHz incident wave is calculated to be 2.12 dB in the range of 40-240 km altitude. The distribution of absorption attenuation coefficients is shown in Fig. 6.

Heating antenna 3 dB beamwidth: 19.5/24@5 MHz, 17/21.8@7 MHz. Based on this, the antenna beam diameter at an altitude of 100 km is approximately 35 km@5 MHz and 33 km@7 MHz. At an altitude of 120 km, the antenna beam diameter is approximately 42 km@5 MHz and 40 km@7 MHz. The ionospheric holes created by the release at 100 km have a diameter of approximately 25 km, while at 120 km, the ionospheric holes have a diameter of approximately 30 km. The scale of the ionospheric holes can cover about 70–80% of the heating antenna beam.

### Radio wave propagation in the modulated ionosphere

In order to further investigate the impact of the ionospheric depletion structure generated by SF_6_ release on the information transmission link, shortwave ray tracing was conducted to simulate the changes in the propagation path and direction of the shortwave signals at different frequencies after traversing the depletion region. Ray tracing is an effective method for studying the ionospheric wave propagation, especially for radio wave signals above the high frequency band. It can accurately describe the propagation paths of electromagnetic waves^30–32^. In this section, a three-dimensional numerical ray tracing method is used to study the propagation characteristics of shortwave signals in the artificial depletion region.

**Figure 7:**
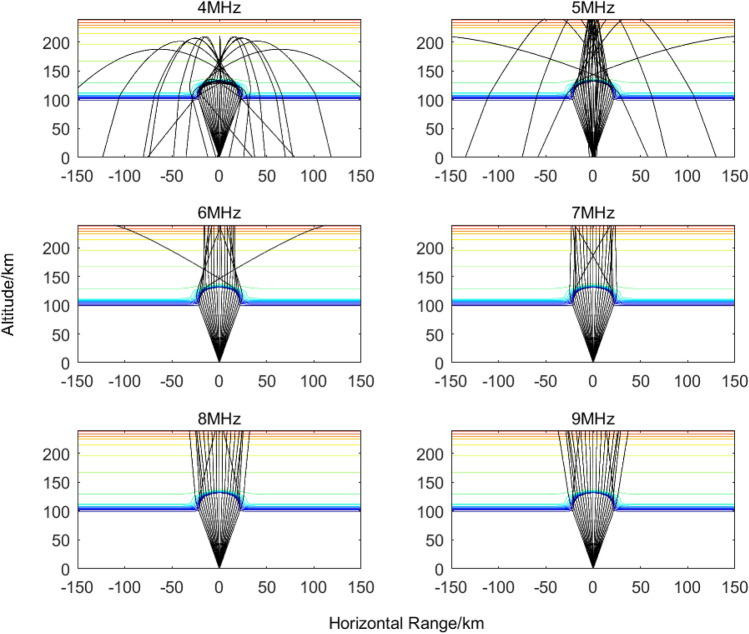
Propagation process of electromagnetic signals at different frequencies after releasing 30 kg SF_6_ at an altitude of 100 km for 1000 s.

**Figure 8:**
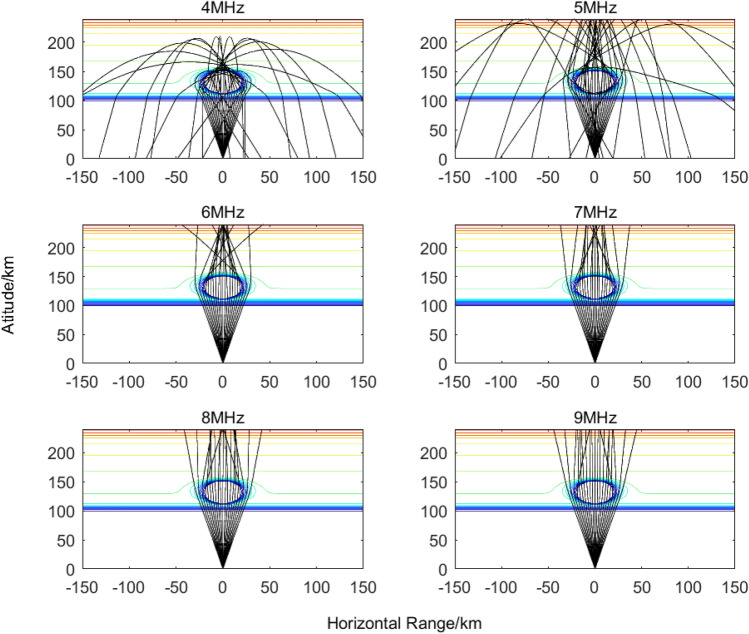
Propagation process of electromagnetic signals at different frequencies after releasing 30 kg SF_6_ at an altitude of 120 km for 1000 s.

To further investigate the propagation characteristics of the heating pump wave beam in controlling the ionosphere, based on the half-power beamwidth of the heating pump wave beam, the short-wave ray tracing results of the 4–9 MHz ionospheric heated pump wave at an altitude of 100 km and 120 km with the release of 30 kg of SF_6_ are given in Figs. 7, 8, respectively, at 1000 s after the release.

From Figs. 7, 8, it can be observed that by releasing electron attachment chemicals such as SF_6_ at low ionospheric altitudes, a large-scale electron-depleted region is formed, which can cover more than 80% of the heating beam area. This not only reduces the absorption attenuation of the heating pump wave but also, due to the formation of a spherical electron-density hole, the refractive index inside the hole is lower than that of the surrounding plasma. As a result, the heating pump wave beam becomes concentrated in a smaller area, leading to increased heating energy density. This is beneficial for generating a focusing heating effect and enhancing the overall heating efficiency.

## Conclusion and discussion

Enhancing the efficiency of ionospheric heating is one of the important research topics in ionospheric heating technology. It is expected that the absorption effect of high-frequency pump wave energy in the low ionosphere can be greatly reduced by releasing neutral gas into the low ionosphere to form ionospheric electron density holes. This paper proposes, for the first time, a method to reduce the absorption of ionospheric heating pump waves by releasing electron attachment chemicals at low ionospheric altitude s to form a large-scale electron density hole. A model for mitigating the absorption of ionospheric heating based on SF_6_ release is established, and the absorption mitigation effects of different frequency pump waves are quantitatively calculated. The propagation characteristics of short-wave signals in ionospheric holes are studied using a three-dimensional ray tracing method, and the results show that the method of releasing electron attachment chemicals not only reduces the absorption attenuation of the heating pump waves but also creates spherical electron density holes that contribute to the generation of a focusing heating effect, thereby enhancing the overall heating efficiency. By combining ionospheric heating and ionospheric chemical release, this study explores approaches and methods to improve the efficiency of ionospheric heating. The results are of great significance for studying the nonlinear interaction between electromagnetic wave and ionospheric plasma and improving the heating efficiency of the ionosphere.

The following conclusions are obtained from theoretical modelling and simulation calculations:

- (1). Releasing SF_6_ gas at an appropriate altitude can significantly reduce the absorption attenuation of high-frequency electromagnetic waves in the low ionosphere by creating ionospheric electron density holes.
- (2). The maximum absorption altitude varies under different ionospheric backgrounds (different locations, seasons, solar activity) and heating frequencies, and the release altitude of the chemical needs to be optimized based on the ionospheric background conditions and heating frequency.
- (3). Due to the small diffusion coefficient of the released chemical, the ionospheric hole scale is small, so it is necessary to control the release location precisely in the space experiment of the low ionospheric chemical release.
- (4). By releasing electron attachment chemicals such as SF6 at low ionospheric altitudes, a large-scale electron-density depletion region is formed. This method reduces the absorption attenuation of the heating pump waves. Additionally, the formation of spherical electron-density hole results in a lower refractive index compared to the surrounding plasma. As a result, the heating pump wave beam becomes concentrated in a smaller area, which can increase the heating energy density. This is beneficial for generating a focusing heating effect and enhancing the overall heating efficiency.

## Data Availability

The international reference ionosphere (IRI) parameters and atmospheric parameters used in this paper are obtained from matlab2014a. And they also can be available through the website (https://ccmc.gsfc.nasa.gov/modelweb/models/iri2016_vitmo.php). Other datasets are available at 10.5281/zenodo.10958526.

## References

[1] Tellegen BDH (1933). Interaction between radio-waves?. Nature.

[2] Utlaut WF, Cohen R (1971). Modifying the ionosphere with intense radio waves. Science.

[3] Utlaut WF (1970). An ionospheric modification experiment using very high power, high frequency transmission. J. Geophys. Res..

[4] Wu J, Wu J, Rietveld MT, Haggstrom I, Zhao H, Xu Z (2017). The behavior of electron density and temperature during ionospheric heating near the fifth electron gyrofrequency. J. Geophys. Res. Space Phys..

[5] Blagoveshchenskaya NF, Borisova TD, Kalishin AS, Egorov IM, Zagorskiy GA (2022). Disturbances of electron density in the high latitude upper (F-region) ionosphere induced by X-mode HF pump waves from EISCAT UHF radar observations. Arct. Antarct. Res..

[6] Blagoveshchenskaya NF, Borisova TD, Yeoman TK, Häggström I, Kalishin AS (2015). Modification of the high latitude ionosphere F region by X-mode powerful HF radio waves: Experimental results from multi-instrument diagnostics. J. Atmos. Sol. Terr. Phys..

[7] Blagoveshchenskaya NF, Borisova TD, Yeoman TK, Rietveld MT, Häggström I, Ivanova IM (2013). Plasma modifications induced by an X-mode HF heater wave in the high latitude F region of the ionosphere. J. Atmos. Sol. Terr. Phys..

[8] Carroll JC, Violette EJ, Utlaut WF (1974). The Platteville high power facility. Radio Sci..

[9] Bernhardt PA, Scales WA, Grach SM (1991). Excitation of artificial airglow by high power radio waves from the “Sura” ionospheric heating facility. Geophys. Res. Lett..

[10] Rodriguez P, Kennedy EJ, Keskinen MJ (1998). The WIND-HAARP Experiment: Initial results of high power radiowave interactions with space plasmas. Geophys. Res. Lett..

[11] Bailey PG, Worthington NC. History and Applications of HAARP Technologies: The High Frequency Active Auroral Research Program[C]//IECEC-97 Proceedings of the Thirty-Second Intersociety Energy Conversion Engineering Conference (Cat. No. 97CH6203). IEEE, 2: 1317–1322. (1997).

[12] Rietveld MT, Kohl H, Kopka H (1993). Introduction to ionospheric heating at Tromsø—I. Experimental overview. J. Atmos. Terr. Phys..

[13] Kohl H, Kopka H, Stubbe P (1993). Introduction to ionospheric heating experiments at Tromsø—II. Scientific problems. J. Atmos. Terr. Phys..

[14] Gordon WE, Carlson HC (1974). Arecibo heating experiments. Radio Sci..

[15] Fejer JA, Gonzales CA, Ierkic HM (1985). Ionospheric modification experiments with the Arecibo heating facility. J. Atmos. Terr. Phys..

[16] Lv L, Zhensen Wu, Li Q, Hao S, Ding J, Ma G, Chen J (2019). Heating frequency optimization for artificial feld-aligned scattering. Plasma Sci. Technol..

[17] Lv L, Zhensen Wu, Li Q, Hao S, Ma G, Yang JuTao, Ding J, Jian Wu (2019). Features of downshifted maximum spectra during a dual-pump ionospheric heating experiment. Adv. Space Res..

[18] Bernhardt PA, Rodriguez P, Siefring CL, Lin CS (1991). Field-aligned dynamics of chemically induced perturbations to the ionosphere. J. Geophys. Res..

[19] Bernhardt PA, Siefring CL, Rodriguez P, Haas DG, Baumback ΜM, Romero HA, Solin DA, Djuth FT, Duncan LM, Hunton DE, Pollock CJ, Sulzer MP, Tepley CA, Wagner LS, Goldstein JA (1995). The ionospheric focused heating experiment. J. Geophys. Res..

[20] Djuth FT, Sulzer MP, Elder JH, Groves KM (1995). The CRRES AA2 release: HF wave-plasma interactions in a dense Ba^+^ cloud. J. Geophys. Res..

[21] Scales WA, Bernhardt PA, Ganguli G (1995). Early time evolution of a chemically produced electron depletion. J. Geophys. Res..

[22] Pedersen T, Gustavsson B, Mishin E, Kendall E, Mills T, Carlson HC, Snyder AL (2010). Creation of artificial ionospheric layers using high-power HF waves. Geophys. Res. Lett..

[23] Zhao H, Xu Z, Wang Y, Xie S, Xue K, Wang C, Wu J, Gao J, Xu Z, Zheng Y (2022). Over-the-horizon channel of radio communication at VHF band via artificial plasma clouds. IEEE Trans. Antennas Propag..

[24] Caton RG (2017). Artifical ionospheric modification: The metal oxide space cloud experiment. Radio Sci..

[25] Zhao ZY, Zhang YN (2011). Ionospheric disturbances produced by chemical releases and the resultant effects on short-wave ionospheric propagation. J. Geophys. Res..

[26] Bernhardt PA (1988). Cross-B convection of artificially created, negative-ion clouds and plasma depressions: Low-speed flow. J. Geophys. Res..

[27] Zhao H, Feng J, Xu Z, Wu Z (2016). A temporal three-dimensional simulation of samarium release in the ionosphere. J. Geophys. Res. Space Phys..

[28] Lee MC, Fejer JA (1978). Theory of short-scale field-aligned density striations due to ionospheric heating. Radio Sci..

[29] Wang X, Zhou C, Liu M (2016). Parametric instability induced by X-mode wave heating at EISCAT. J. Geophys. Res. Space Phys..

[30] Haselgrove J. Ray theory and a new method of ray tracing. In *Physics of the Ionosphere*. 355–360 (Cavendish Laboratory, Cambridge, London, 1955).

[31] Kelso JM (1968). Ray tracing in the ionosphere. Radio Sci..

[32] Lin F, Chen J, Ding G, Jiao Y (2023). Short-term fine-grained regional MUF prediction for HF communication based on time-series decomposition. IEEE Trans. Antennas Propag..

